# Tumor hypoxia is associated with global copy-number alteration burden and subtype-dependent overall survival in breast cancer: Evidence from TCGA and METABRIC

**DOI:** 10.1371/journal.pone.0350829

**Published:** 2026-06-02

**Authors:** Wenhan Yang

**Affiliations:** College of Arts and Sciences, Georgia State University, Atlanta, Georgia, United States of America; Weill Cornell University, UNITED STATES OF AMERICA

## Abstract

Tumor hypoxia is biologically important in breast cancer, but its prognostic value may be distorted by intrinsic molecular subtype composition. This study evaluated whether hypoxia-related prognosis was subtype-dependent and whether hypoxia was associated with genome-wide copy-number alteration (CNA) burden. Transcriptome-derived hypoxia scores, CNA burden, and overall survival data were analyzed from TCGA and METABRIC. Survival differences between hypoxia groups were assessed using Kaplan–Meier analysis and log-rank tests. Multivariable Cox models were used to evaluate hypoxia-related prognosis after adjustment for subtype and eligible clinical covariates. Proportional hazards diagnostics and Weibull accelerated failure time models were further applied to address potential model-assumption violations. In TCGA, the cohort-wide survival association was no longer evident after adjustment for subtype and clinical covariates. The clearest subtype-specific signal was observed in Luminal B tumors. Within this subtype, low hypoxia was associated with better survival after adjustment for age, stage, and CNA burden. In METABRIC, high hypoxia remained associated with poorer survival in Weibull accelerated failure time models. Higher hypoxia was also consistently associated with greater CNA burden across both cohorts. These findings support subtype-aware interpretation of hypoxia biomarkers and suggest a reproducible link between hypoxia and genomic instability in breast cancer.

## Introduction

Hypoxia is a common feature of solid tumors [1–3]. It arises when tumor growth outpaces oxygen delivery and when tumor microcirculation is structurally or functionally abnormal [2,3]. In breast cancer, hypoxic stress is not merely a passive consequence of tumor expansion. It has been linked to malignant progression and treatment resistance [2,5]. These effects are partly mediated by hypoxia-inducible factor signaling [3,4]. They also involve angiogenesis, metabolic adaptation, invasion, and stem-like phenotypes [3,4,6]. Many of these processes are mediated through HIF-1α-regulated transcriptional programs, and clinical studies have associated higher HIF-1α levels with worse breast cancer outcomes [7]. At the transcriptomic level, hypoxia can be assessed using expression-based signatures. These signatures capture coordinated cellular responses to hypoxia [8]. Hypoxia-related expression signatures have also shown prognostic relevance across multiple cancer types, including breast cancer [8,9].

Breast cancer, however, is biologically heterogeneous rather than a single disease entity. Intrinsic molecular subtypes—including Luminal A, Luminal B, Basal-like, and HER2-enriched—differ in prognosis, transcriptional state, and genomic architecture [10–13]. This heterogeneity has important implications for survival analysis. A cohort-wide association between hypoxia and outcome may be distorted if particular subtypes are overrepresented in one hypoxia group. Hypoxia should therefore be interpreted in a subtype-aware framework rather than through pooled comparisons alone.

Genomic instability provides a complementary perspective on hypoxia-associated tumor biology. Copy-number alterations (CNAs) and broader chromosomal instability are common in breast cancer and can be summarized using genome-wide burden measures [14,15]. Experimental and translational studies suggest a biologically plausible link between hypoxic stress and impaired DNA repair [16]. In hypoxic cancer cells, RAD51 is downregulated and homologous recombination activity is reduced [16]. Together, these observations suggest that transcriptomic hypoxia may co-occur with global CNA burden in breast tumors. They also raise the possibility that this relationship contributes to subtype-specific survival patterns.

To examine these questions, this study integrates transcriptome-derived hypoxia measures and genome-wide CNA burden across TCGA and METABRIC, two large breast cancer cohorts harmonized in cBioPortal format [17–22]. Hypoxia and CNA burden were defined differently across the two cohorts. Therefore, the analysis focused on consistency in the direction of associations and on subtype-aware interpretation. Absolute values and effect sizes were not directly compared across cohorts. The study was designed to address four related aims. First, we tested whether hypoxia was associated with overall survival after accounting for intrinsic subtype. Second, we examined whether cohort-wide hypoxia grouping in TCGA was driven by subtype composition and whether subtype-specific analyses revealed heterogeneous associations. Third, we evaluated whether hypoxia was associated with CNA burden and whether CNA burden contributed additional prognostic information after multivariable adjustment. Fourth, we explored driver-gene enrichment within TCGA Luminal B tumors.

## Materials and Methods

### Data sources and study design

This study analyzed clinical and molecular data from TCGA-BRCA and METABRIC obtained as harmonized cBioPortal tables [17 21]. TCGA-BRCA was treated as the discovery cohort [17]. It provided clinical annotations, supplementary BUFFA hypoxia scores, continuous gene-level log2 CNA data, and somatic mutation calls. METABRIC was treated as the external validation cohort [18,19]. It provided clinical annotations, Illumina microarray expression data, and discrete gene-level CNA calls. These CNA calls were coded as −2, −1, 0, 1, and 2. Hypoxia and CNA burden were derived differently across the two cohorts. Therefore, cross-cohort comparisons were interpreted mainly in terms of directional consistency. Subtype-aware patterns were emphasized, rather than direct numerical comparisons of absolute values or effect sizes.

**Fig 1:**
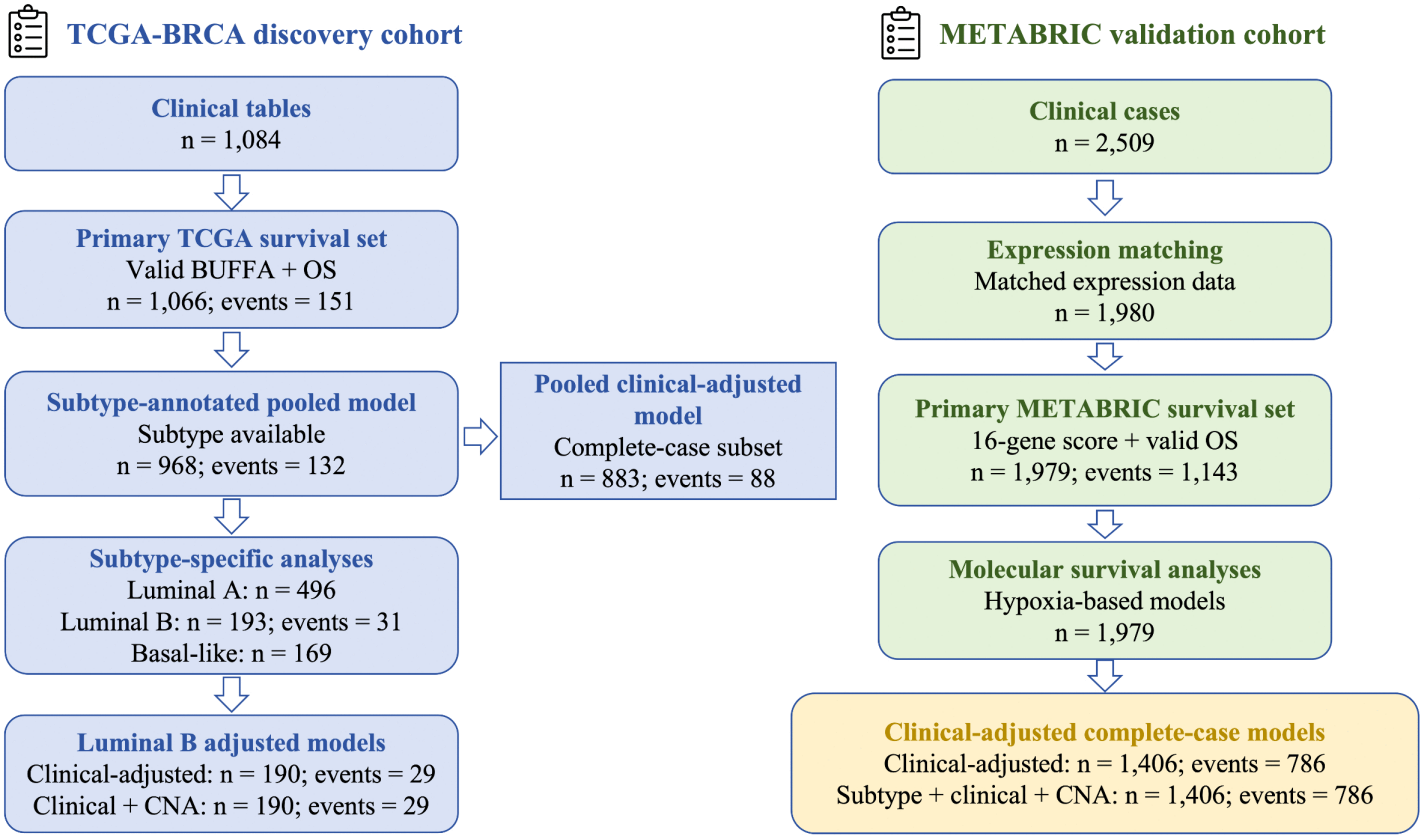
Analysis sample flow across the TCGA discovery cohort and the METABRIC validation cohort. TCGA was used as the discovery cohort and METABRIC as the validation cohort. Boxes show the numbers of cases retained at each analytic step, including complete-case subsets used for adjusted survival models.

The overall study design and analytic sample flow are summarized in Fig 1. In TCGA, 1,084 cases were available in the clinical tables. Of these, 1,066 patients had non-missing BUFFA hypoxia scores and valid overall survival (OS) data. This group included 151 events and formed the primary TCGA survival set. Among these, 968 also had intrinsic subtype annotations and were included in the pooled subtype-annotated analysis set. Subtype-specific analyses were then performed in Luminal A (n = 496), Luminal B (n = 193; 31 events), and Basal-like tumors (n = 169). The same TCGA Luminal B subset was used in all downstream subtype-specific analyses. These analyses included adjusted Luminal B survival models, CNA-related analyses, and mutation-related analyses. This approach maintained a consistent subtype-defined analytic population.

For pooled multivariable analyses in TCGA, complete-case analysis was used when clinical covariates were required. The pooled clinical-adjusted model included 883 patients with 88 events. In the subtype-specific Luminal B analyses, the clinical-adjusted and clinical-plus-CNA models were both fitted in a complete-case subset of 190 patients with 29 events.

In METABRIC, 2,509 clinically annotated cases were available. Among these, 1,980 had matched expression data. A total of 1,979 patients had a computed 16-gene hypoxia score and valid OS data. This set included 1,143 events. It was used as the primary METABRIC survival set and as the unadjusted molecular survival analysis set. For multivariable METABRIC analyses incorporating clinical covariates, complete-case analysis was again used. The final clinical-adjusted model and the subtype-plus-clinical-plus-CNA model were both fitted in 1,406 patients with 786 events.

Model-specific sample sizes are reported separately to distinguish the broader descriptive cohorts from the smaller complete-case subsets used in adjusted survival models. A summary of adjusted survival models, covariates included, complete-case sample sizes, and event counts is provided in S1 Table.

### Data Preprocessing, Harmonization, and Variable Construction

Clinical patient-level and sample-level tables were merged using standardized PATIENT_ID and SAMPLE_ID fields. Comment lines and metadata rows in cBioPortal-formatted text files were removed before import [20,21]. OS time was extracted directly from the cohort-specific clinical survival field and was recorded in months in both cohorts. Vital status was recoded as a binary event indicator (death vs censored). Samples were excluded from a given analysis if they had missing OS time, non-positive follow-up time, or missing values for the molecular variable required for that specific analysis.

Hypoxia was defined in a cohort-specific but conceptually aligned manner. In TCGA, the BUFFA hypoxia score was analyzed both as a continuous covariate and as a dichotomized variable. The continuous score was used in the pooled subtype-adjusted model to preserve score ordering. This approach also avoided unnecessary information loss. Dichotomized hypoxia groups were used for Kaplan–Meier visualization and subtype-specific comparisons. Two dichotomization strategies were applied in TCGA: a cohort-wide median split for pooled descriptive analyses and within-subtype median splits for subtype-specific analyses. In METABRIC, a 16-gene hypoxia score was computed as the mean of gene-wise z-scored expression values across ALDOA, ANGPTL4, CA9, ENO1, HK2, LDHA, PGK1, SLC2A1, VEGFA, PDK1, ADM, BNIP3, NDRG1, PFKFB3, EGLN1, and EGLN3 [8,9]. Patients without an expression-derived hypoxia score were excluded from METABRIC hypoxia-based analyses.

CNA burden was summarized at the sample level from gene-level CNA data. In TCGA, continuous log2 CNA values were summarized as the mean absolute log2 CNA across genes. As an additional descriptive measure, the proportion of genes with |log2 CNA| ≥ 0.2 was also calculated. In METABRIC, discrete CNA calls ranging from −2–2 were summarized as the mean absolute discrete CNA call across genes. As a secondary descriptive measure, the proportion of genes with any non-neutral CNA call (CNA ≠ 0) was also calculated. Because the underlying CNA scales differed between TCGA and METABRIC, CNA burden was interpreted within cohort and was not treated as directly comparable on an absolute scale across cohorts.

Intrinsic subtype indicators were taken from the harmonized cohort-level clinical tables. Standard clinical covariates were harmonized separately within each cohort. Adjusted survival models used complete-case analysis for the variables included in each model. Processed per-sample variables generated in this study included hypoxia measures, CNA-burden summaries, survival analysis variables, and model-ready analytic tables for the final complete-case analyses.

Somatic mutation analyses were conducted in TCGA using nonsynonymous variants only. For Luminal B driver-event enrichment, sample-level mutation indicators were created for a prespecified panel of recurrent breast cancer driver genes [14]. Mutation frequencies were then compared between the high- and low-hypoxia Luminal B groups in downstream analyses.

### Statistical Modeling and Inference Strategy

Overall survival (OS) was summarized using Kaplan–Meier estimators and compared between hypoxia groups using log-rank tests [23,24]. The principal regression framework in TCGA was the Cox proportional hazards model [25]. Standard clinical covariates were prespecified as candidate confounders, including age, tumor stage, grade, and treatment variables. Covariates were included in a given model only when they were available and sufficiently complete in the corresponding analytic dataset. In pooled TCGA analyses, the clinical-adjusted model included the standardized continuous BUFFA hypoxia score, intrinsic subtype, and the eligible clinical covariates. In subtype-specific TCGA analyses, particularly in Luminal B, parsimonious clinical adjustment was used because of the limited number of events. The main Luminal B model adjusted for age and stage, whereas treatment and CNA burden were evaluated in sensitivity or extension models.

The general Cox proportional hazards model was defined in Eq (1):


$$\begin{array}{c} {\text{h}}_{\text{i}}(\text{t})={\text{h}}_{\text{0}}(\text{t})\text{exp}\left(\eta_{\text{i}}\right) \end{array}$$ (1)

where $\;{\text{h}}_{\text{i}}(\text{t})\text{ i}$ s the hazard for patient i at time t, ${\text{h}}_{\text{0}}(\text{t})\;$ is the unspecified baseline hazard, and $\eta_{\text{i}}$ is the linear predictor.

For the pooled TCGA subtype-adjusted model, the linear predictor in Eq (1) was specified as Eq (2):


$$\begin{array}{c} \eta_{\text{i}}=\beta_{\text{1}}\text{BUFF}{\text{A}}_{\text{z},\text{i}}+\overset{⊤}{\underset{\text{2}}{\beta }}{\text{S}}_{\text{i}} \end{array}$$ (2)

where $\;\text{BUFF}{\text{A}}_{\text{z},\text{i}}\;$ denotes the globally standardized BUFFA hypoxia score for patient i, and ${\text{S}}_{\text{i}}$ denotes the intrinsic subtype indicator vector. For the pooled TCGA clinical-adjusted model, the linear predictor was extended as Eq (3):


$$\begin{array}{c} \eta_{\text{i}}=\beta_{\text{1}}\text{BUFF}{\text{A}}_{\text{z},\text{i}}+\overset{⊤}{\underset{\text{2}}{\beta }}{\text{S}}_{\text{i}}+\overset{⊤}{\underset{\text{3}}{\beta }}{\text{Z}}_{\text{i}} \end{array}$$ (3)

where ${\text{Z}}_{\text{i}}$ denotes the eligible standard clinical covariates in the final pooled TCGA analytic dataset.

For Kaplan–Meier visualization and subtype-specific TCGA analyses, hypoxia was dichotomized as low versus high, with high hypoxia treated as the reference group. The hypoxia indicator was defined as Eq (4):


$$\begin{array}{c} {\text{H}}_{\text{i}}=\left\{ \begin{array}{c} @l\text{1},\text{ if patient i belonged to the low}-\text{hypoxia group}\\ \text{0},\text{ if patient i belonged to the high}-\text{hypoxia group} \end{array}\right.\; \end{array}$$ (4)

Within a given TCGA subtype, the base model was defined as Eq (5):


$$\begin{array}{c} {\text{h}}_{\text{i}}(\text{t})={\text{h}}_{\text{0}}(\text{t})\;\text{exp}\left(\beta_{\text{1}}{\text{H}}_{\text{i}}\right) \end{array}$$ (5)

In TCGA Luminal B, the parsimonious clinical-adjusted main model was specified as Eq (6):


$$\begin{array}{c} {\text{h}}_{\text{i}}(\text{t})={\text{h}}_{\text{0}}(\text{t})\;\text{exp}\left(\beta_{\text{1}}{\text{H}}_{\text{i}}+\beta_{\text{2}}\text{AG}{\text{E}}_{\text{i}}+\beta_{\text{3}}\text{STAGE}{\text{2}}_{\text{i}}\right) \end{array}$$ (6)

where $\text{STAGE}{\text{2}}_{\text{i}}\;$ represents the dichotomized stage variable (I/II vs III/IV).

A treatment sensitivity model in TCGA Luminal B was specified as Eq (7):


$$\begin{array}{c} {\text{h}}_{\text{i}}\left(\text{t}\right)={\text{h}}_{\text{0}}\left(\text{t}\right)\text{exp}\left(\beta_{\text{1}}{\text{H}}_{\text{i}}+\beta_{\text{2}}\text{AG}{\text{E}}_{\text{i}}+\beta_{\text{3}}\text{STAGE}{\text{2}}_{\text{i}}+\beta_{\text{4}}\text{TREA}{\text{T}}_{\text{i}}\right) \end{array}$$ (7)

where ${\text{TREAT}}_{\text{i}}$ denotes the binary treatment indicator.

The TCGA Luminal B CNA extension model was specified as Eq (8):


$$\begin{array}{c} {\text{h}}_{\text{i}}\left(\text{t}\right)={\text{h}}_{\text{0}}\left(\text{t}\right)\text{exp}\left(\beta_{\text{1}}{\text{H}}_{\text{i}}+\beta_{\text{2}}\text{AG}{\text{E}}_{\text{i}}+\beta_{\text{3}}\text{STAGE}{\text{2}}_{\text{i}}+\beta_{\text{4}}\text{CN}{\text{A}}_{\text{IQR},\text{i}}\right) \end{array}$$ (8)

where $\text{CN}{\text{A}}_{\text{IQR},\text{i}}\;$ denotes the sample-level CNA burden entered on an interquartile-range-scaled scale.

In METABRIC, a 16-gene hypoxia score was computed from the mean of gene-wise z-scored expression values across the predefined gene panel:


$$\begin{array}{c} \text{HYPOXI}{\text{A}}_{\text{i}}=\frac{\text{1}}{\text{16}}\sum_{\text{g}=\text{1}}^{\text{16}}{\text{z}}_{\text{ig}} \end{array}$$ (9)

where ${\text{z}}_{\text{ig}}$ is the within-cohort standardized expression value of gene g in sample i.

For binary survival analyses in METABRIC, patients were divided into low- and high-hypoxia groups using the cohort-wide median 16-gene hypoxia score. High hypoxia was used as the reference group. The adjusted METABRIC Cox model was specified as Eq (10):


$$\begin{array}{c} {\text{h}}_{\text{i}}(\text{t})={\text{h}}_{\text{0}}(\text{t})\;\text{exp}\left(\beta_{\text{1}}{\text{H}}_{\text{i}}+\overset{⊤}{\underset{\text{2}}{\beta }}{\text{S}}_{\text{i}}+\overset{⊤}{\underset{\text{3}}{\beta }}{\text{Z}}_{\text{i}}\right) \end{array}$$ (10)

where ${\text{H}}_{\text{i}}$ denotes the low-versus-high hypoxia indicator, ${\text{S}}_{\text{i}}$ denotes subtype indicators, and ${\text{Z}}_{\text{i}}$ denotes the standard clinical covariates age, stage, grade, and treatment.

The METABRIC CNA-augmented model was written as Eq (11):


$$\begin{array}{c} {\text{h}}_{\text{i}}(\text{t})={\text{h}}_{\text{0}}(\text{t})\text{exp}\left(\beta_{\text{1}}{\text{H}}_{\text{i}}+\overset{⊤}{\underset{\text{2}}{\beta }}{\text{S}}_{\text{i}}+\overset{⊤}{\underset{\text{3}}{\beta }}{\text{Z}}_{\text{i}}+\beta_{\text{4}}\text{CN}{\text{A}}_{\text{i}}\right) \end{array}$$ (11)

where ${\text{CNA}}_{\text{i}}$ denotes the sample-level mean absolute discrete CNA burden.

The proportional hazards (PH) assumption was assessed for each Cox model using Schoenfeld residual tests and graphical diagnostics. Weibull accelerated failure time (AFT) models were fitted when key METABRIC Cox models violated the proportional hazards (PH) assumption. Their time ratios were used as the preferred effect estimates for interpretation. The general Weibull AFT model was written as Eq (12):


$$\begin{array}{c} \text{log}\left({\text{T}}_{\text{i}}\right)=\mu +\alpha_{\text{1}}{\text{H}}_{\text{i}}+\overset{⊤}{\underset{\text{2}}{\alpha }}{\text{S}}_{\text{i}}+\overset{⊤}{\underset{\text{3}}{\alpha }}{\text{Z}}_{\text{i}}+\alpha_{\text{4}}\text{CN}{\text{A}}_{\text{i}}+\sigma \epsilon_{\text{i}} \end{array}$$ (12)

where ${\text{T}}_{\text{i}}$ denotes survival time for patient i, μ is the intercept, σ is the scale parameter, and $\epsilon_{\text{i}}$ follows the extreme-value distribution implied by the Weibull AFT parameterization. Exponentiated coefficients from the AFT model were interpreted as time ratios (TRs), where TR=exp(α)  indicates longer survival time associated with the corresponding covariate level.

Pearson’s chi-square test was used to assess the association between pooled TCGA hypoxia grouping and intrinsic subtype distribution. Wilcoxon rank-sum tests compared CNA burden between hypoxia groups. Fisher’s exact tests were used for Luminal B driver-gene enrichment analyses, and Benjamini–Hochberg false discovery rate correction was applied across genes [26]. All hypothesis tests were two-sided unless otherwise stated. Detailed proportional hazards diagnostic results for Cox models are provided in S2 Table.

## Results

### Cohort-wide hypoxia grouping is confounded by intrinsic subtype in TCGA

**Fig 2:**
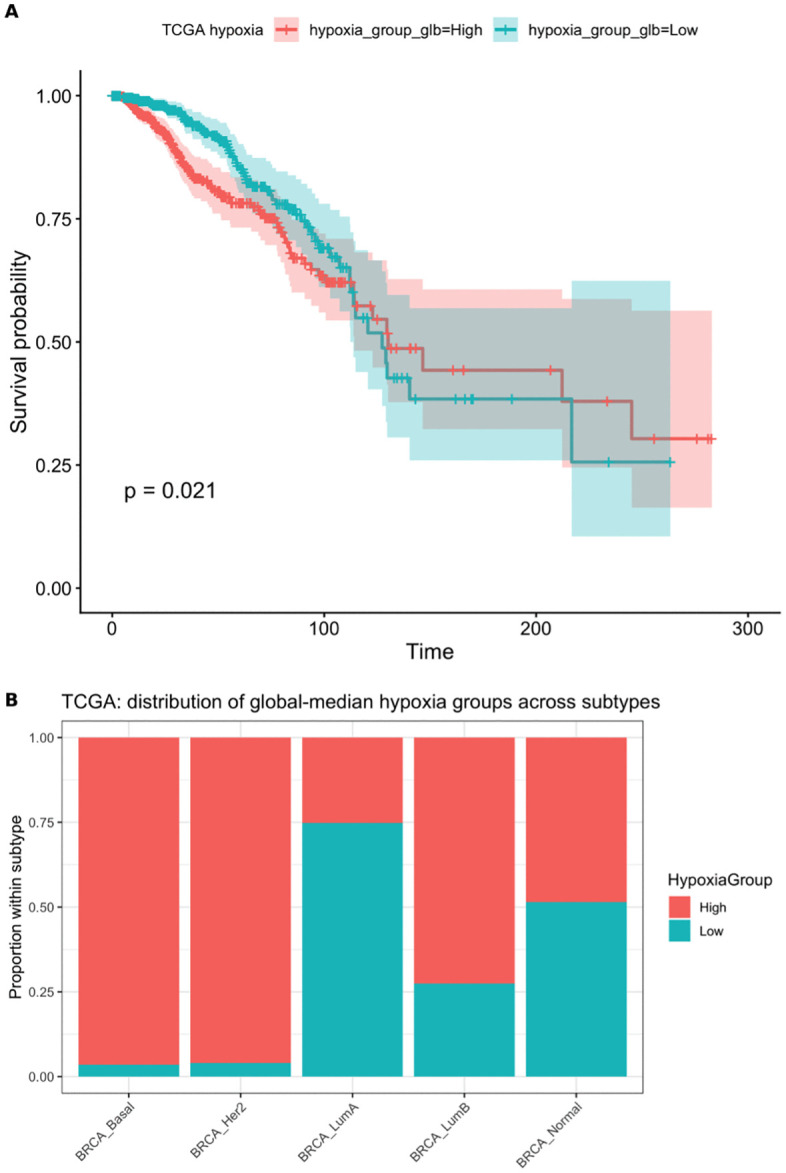
TCGA pooled analyses. **(A)** Kaplan-Meier overall survival in TCGA-BRCA by hypoxia group defined using the cohort-wide median BUFFA score (n = 1066; events = 151). Shaded bands denote 95% confidence intervals. **(B)** Distribution of global-median hypoxia groups across intrinsic subtypes in TCGA-BRCA. Bars show within-subtype proportions; association tested by Pearson’s chi-square test (p < 2.2 × 10^-16).

In TCGA-BRCA, 1,066 patients had non-missing BUFFA hypoxia scores and valid overall survival (OS) data, including 151 deaths. When patients were dichotomized using the cohort-wide median BUFFA hypoxia score, Kaplan-Meier analysis showed an apparent OS difference between the high- and low-hypoxia groups (Fig 2A; log-rank p = 0.021).

However, the cohort-wide median split produced marked subtype imbalance (Table 1; Pearson’s chi-square p < 2.2 × 10^-16). Most Basal-like and HER2-enriched tumors were classified as high hypoxia, whereas Luminal A tumors were predominantly classified as low hypoxia (Fig 2B). These findings indicate that the pooled TCGA hypoxia signal is strongly confounded by intrinsic subtype composition.

**Table 1:** TCGA-BRCA cross-tabulation of cohort-wide median hypoxia groups by intrinsic subtype.

Subtype	High (global median)	Low (global median)	% High within subtype
Basal-like	163	6	96.4
HER2-enriched	72	3	96.0
Luminal A	125	371	25.2
Luminal B	140	53	72.5
Normal-like	17	18	48.6

Percentages are shown within subtype; association tested by Pearson’s chi-square test (p < 2.2 × 10^-16).

### Subtype-stratified survival analyses identify a Luminal B–specific hypoxia signal

Consistent with the marked subtype imbalance, the subtype-adjusted pooled TCGA Cox model showed no statistically significant association between the continuous BUFFA hypoxia score and OS. The score was modeled per 1 SD increase. The estimated HR was 1.098 (95% CI 0.844–1.428; p = 0.4865; Table 2). Schoenfeld residual diagnostics suggested possible non-proportionality for the BUFFA term in this subtype-adjusted model (global p = 0.0978; BUFFA-term p = 0.0114). Accordingly, this model was treated as subtype-aware descriptive evidence rather than the primary adjusted inferential model.

**Table 2:** Key survival models for overall survival in TCGA and METABRIC.

Cohort/ subtype	Model contrast	n	Events	Estimate	95% CI	p
TCGA (pooled)	Cox: BUFFA per 1 SD (adjusted for subtype)	968	132	HR = 1.10	0.84–1.43	0.487
TCGA (pooled)	Cox: BUFFA per 1 SD (adjusted for subtype + clinical covariates)	883	88	HR = 1.23	0.88–1.72	0.233
TCGA Luminal A	Cox: low vs high hypoxia (within-subtype median)	496	57	HR = 1.31	0.77–2.21	0.32
TCGA Luminal B	Cox: low vs high hypoxia (within-subtype median)	193	31	HR = 0.30	0.14–0.67	0.00294
TCGA Basal-like	Cox: low vs high hypoxia (within-subtype median)	169	22	HR = 1.66	0.71–3.90	0.244
METABRIC	Weibull AFT: low vs high hypoxia (clinical-adjusted)	1406	786	TR = 1.21	1.08–1.36	7.44e-04
METABRIC	Weibull AFT: low vs high hypoxia (subtype + clinical + CNA adjusted)	1406	786	TR = 1.20	1.07–1.34	1.79e-03

Note: Effect estimates are reported as hazard ratios (HRs) for Cox models and time ratios (TRs) for Weibull accelerated failure time (AFT) models; the TCGA BUFFA hypoxia score was standardized per 1 SD in the continuous pooled models.

In the pooled TCGA model additionally adjusted for subtype and available clinical covariates, the BUFFA hypoxia score also remained non-significant (HR = 1.226, 95% CI 0.877–1.715; p = 0.233; n = 883, 88 events). For this clinical-adjusted pooled model, Schoenfeld residual diagnostics did not indicate a clear proportional hazards violation (global p = 0.153; BUFFA-term p = 0.084). Overall, these pooled analyses suggest that the apparent cohort-wide survival association did not persist after adjustment for subtype and eligible clinical covariates.

**Fig 3:**
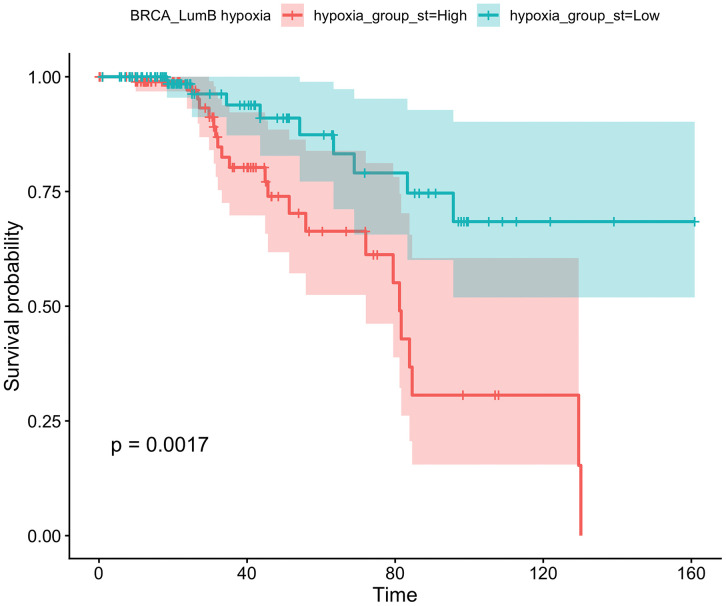
Kaplan–Meier overall survival within Luminal B tumors in TCGA-BRCA. Hypoxia groups were defined using the Luminal B–specific median BUFFA score (n = 193; 31 events). Shaded bands denote 95% confidence intervals.

After stratification by subtype, the clearest survival separation was observed in Luminal B tumors. In TCGA Luminal B (n = 193; 31 deaths; high = 104, low = 89), the low-hypoxia group had better OS than the high-hypoxia group (Fig 3; log-rank p = 0.0017). The corresponding Cox estimate showed the same direction and magnitude (low vs high: HR = 0.303, 95% CI 0.138–0.666; p = 0.00294; Table 2).

This association remained statistically significant in the parsimonious clinical-adjusted Luminal B model including age and stage (HR = 0.329, 95% CI 0.140–0.771; p = 0.0106; n = 190, 29 events). Schoenfeld residual diagnostics did not indicate violation of the proportional hazards assumption for this model (global p = 0.965; hypoxia-term p = 0.893). Because this subtype-specific analysis was based on a limited number of events, the magnitude of effect should be interpreted cautiously, although the direction of association was stable across the Luminal B models. By contrast, Luminal A and Basal-like tumors showed wider confidence intervals and no statistically significant Cox association (Table 2).

### In TCGA Luminal B, higher hypoxia is associated with greater CNA burden and remains associated with OS after CNA adjustment

**Fig 4:**
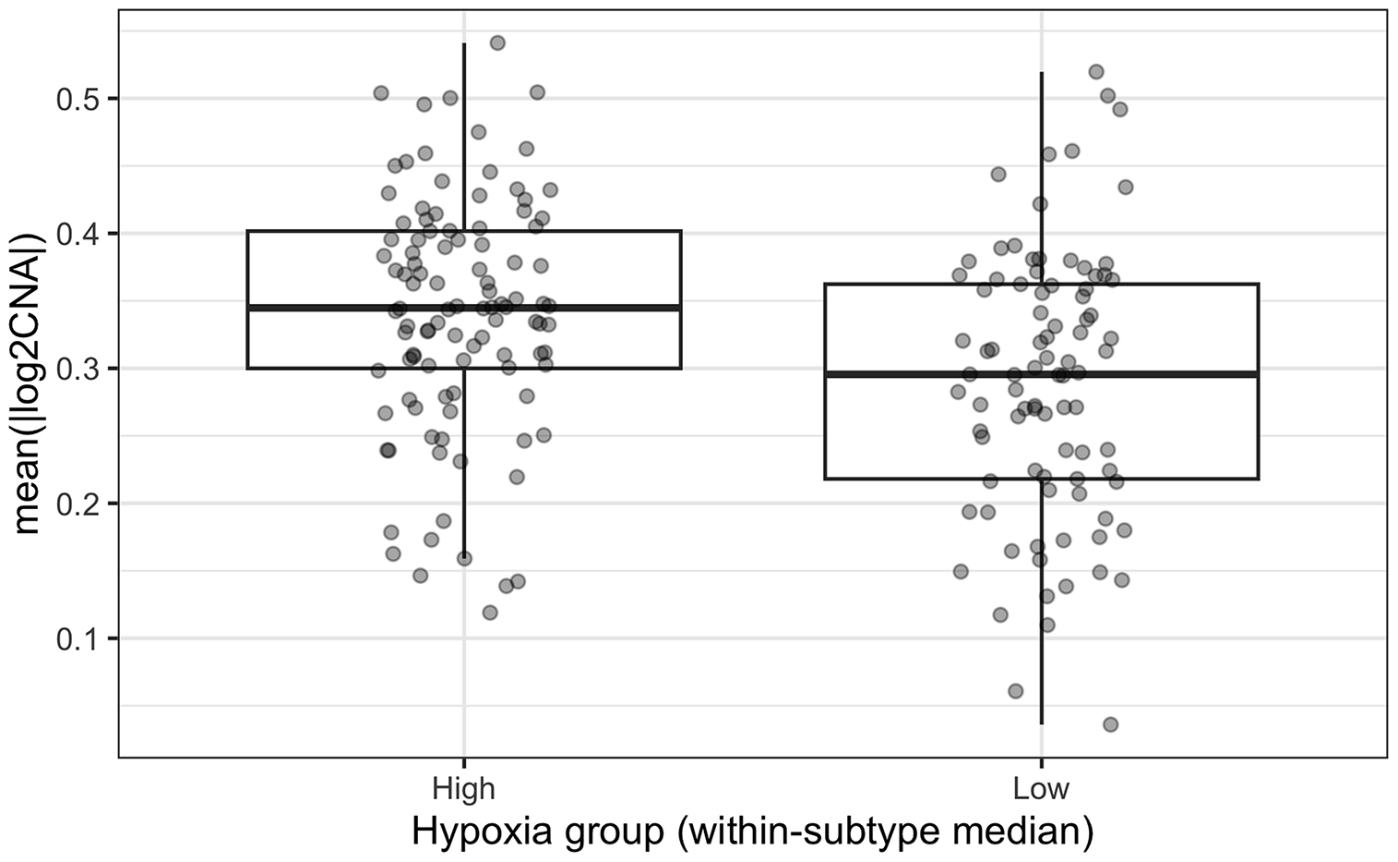
Copy-number alteration (CNA) burden in TCGA Luminal B tumors by within-subtype hypoxia group. Group difference tested by Wilcoxon rank-sum test (p = 2.20 × 10^-4).

Given the clearer Luminal B survival signal, TCGA Luminal B tumors were further evaluated for co-occurrence with genomic instability. CNA burden was higher in the high-hypoxia group than in the low-hypoxia group (Fig 4; Wilcoxon p = 0.00022), indicating an association between hypoxia and greater genome-wide copy-number disruption in this subtype.

The Luminal B extension model included age, stage, and CNA burden as covariates. Low hypoxia remained associated with better OS (HR = 0.360, 95% CI 0.152–0.855; p = 0.0206; Table 3). In contrast, CNA burden itself was not independently associated with OS (HR = 1.398, 95% CI 0.780–2.506; p = 0.26; Table 3). Thus, the TCGA Luminal B data support co-occurrence between higher hypoxia and higher CNA burden, but they do not establish CNA burden as an independent prognostic factor in this subtype-specific analysis. Given the limited event count in Luminal B, this extension model should be interpreted cautiously.

**Table 3:** TCGA Luminal B associations among within-subtype hypoxia group, CNA burden, and overall survival.

TCGA LumB (n = 193)	Result
CNA burden difference (high vs low hypoxia)	Wilcoxon p = 0.00022
Cox base model (hypoxia only): low vs high hypoxia	HR = 0.303 (95% CI 0.138–0.666), p = 0.00294
Cox clinical-adjusted model (age + stage): low vs high hypoxia	HR = 0.329 (95% CI 0.140–0.771), p = 0.0106
Cox clinical + CNA model: low vs high hypoxia	HR = 0.360 (95% CI 0.152–0.855), p = 0.0206
Cox clinical + CNA model: CNA burden (IQR-scaled)	HR = 1.398 (95% CI 0.780–2.506), p = 0.26
Cox treatment sensitivity model (age + stage + any treatment; n = 173, events = 19): low vs high hypoxia	HR = 0.285 (95% CI 0.093–0.878), p = 0.0287

Across the base, clinical-adjusted, CNA-extended, and treatment sensitivity Luminal B Cox models, the direction of the hypoxia association remained stable, whereas the evidence for an independent CNA effect did not. In the treatment sensitivity model additionally adjusted for age, stage, and any treatment, low hypoxia remained associated with better OS (HR = 0.285, 95% CI 0.093–0.878; p = 0.0287; Table 3). Because this model had fewer complete cases and events, it was interpreted as supportive sensitivity evidence rather than as the primary Luminal B model.

### TP53 alteration enrichment in hypoxia-high Luminal B tumors

Driver-gene enrichment analysis in TCGA Luminal B identified TP53 as the only prespecified gene that remained significant after multiple-testing correction. TP53 mutations were enriched in the high-hypoxia group (Table 4; OR = 3.86, 95% CI 2.04–7.32; p = 2.21 × 10^-5; FDR = 2.65 × 10^-4), whereas no other candidate driver gene remained significant after false-discovery-rate adjustment.

**Table 4:** TCGA Luminal B driver-gene enrichment by within-subtype hypoxia group.

Gene	High mut (n = 104)	Low mut (n = 89)	OR (High vs Low)	95% CI	p	FDR
TP53	52/104	18/89	3.86	2.04–7.32	2.21e-05	2.65e-04
PTEN	8/104	3/89	2.18	0.61–7.82	0.229	1
MAP3K1	2/104	5/89	0.37	0.08–1.72	0.252	1
GATA3	24/104	15/89	1.46	0.72–2.98	0.369	1
FGFR1	2/104	0/89	4.37	0.21–92.15	0.500	1
CDH1	5/104	3/89	1.37	0.35–5.38	0.728	1
RB1	4/104	4/89	0.85	0.22–3.24	1	1
NF1	3/104	2/89	1.21	0.23–6.27	1	1
AKT1	2/104	2/89	0.85	0.14–5.04	1	1
ERBB2	1/104	1/89	0.86	0.09–8.37	1	1
CCND1	0/104	0/89	0.86	0.02–43.61	1	1
PIK3CA	31/104	26/89	1.03	0.55–1.90	1	1

Note: Odds ratios were estimated for mutation enrichment in the high-hypoxia group; statistical testing and multiple-testing correction are described in Methods.

### External validation in METABRIC supports the hypoxia-CNA association and an adverse survival association of high hypoxia

**Fig 5:**
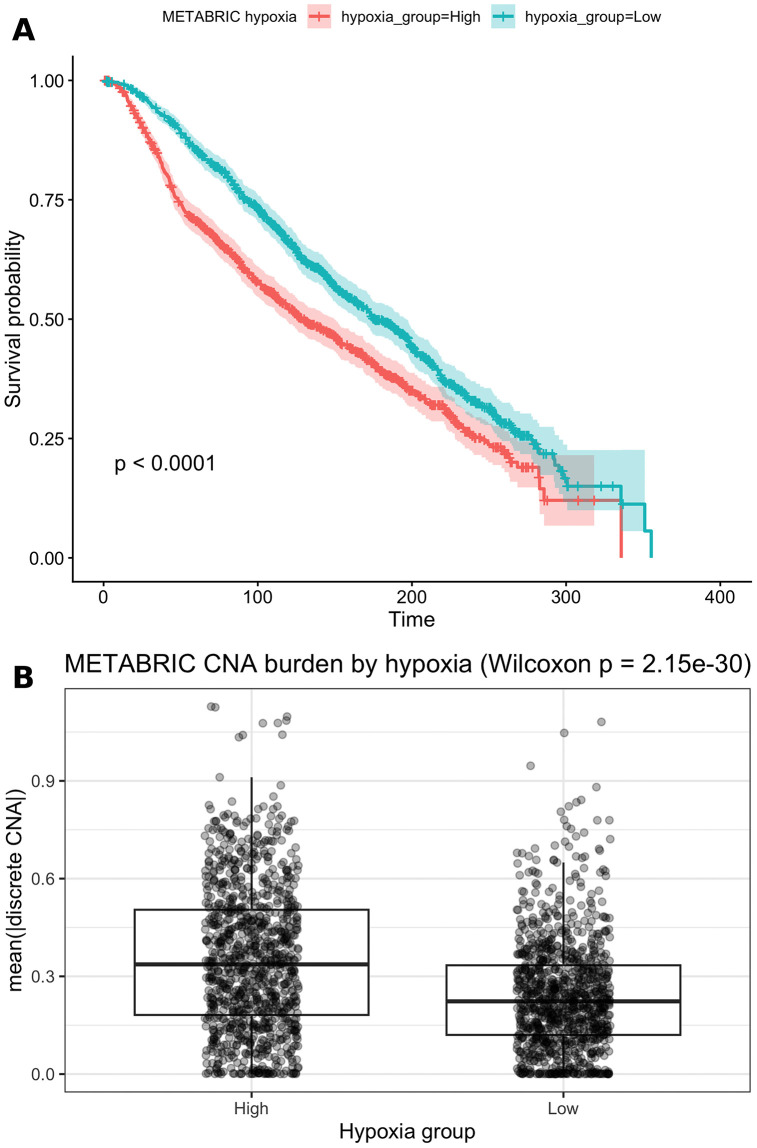
METABRIC validation analyses. **(A)** Kaplan–Meier overall survival by hypoxia group defined using the cohort-wide median 16-gene hypoxia score. **(B)** CNA burden in METABRIC by hypoxia group; CNA burden is summarized as mean(|discrete CNA|) across genes, with Wilcoxon p = 2.15 × 10^-30.

Replication analyses in METABRIC used an independent 16-gene hypoxia score. Descriptive Kaplan-Meier analysis showed worse survival in the high-hypoxia group (Fig 5A). High hypoxia was also associated with higher CNA burden (Fig 5B; Wilcoxon p = 2.15 × 10^-30).

However, Schoenfeld residual diagnostics indicated strong violation of the proportional hazards assumption in the key adjusted METABRIC Cox models (clinical-adjusted Cox: global p = 9.07 × 10^-27, hypoxia-term p = 2.46 × 10^-6; subtype-, clinical-, and CNA-adjusted Cox: global p = 4.34 × 10^-26, hypoxia-term p = 2.65 × 10^-6). Therefore, Weibull accelerated failure time models were used as the preferred framework for interpretation. In the clinical-adjusted model, the low-hypoxia group showed longer survival time than the high-hypoxia group (TR = 1.213, 95% CI 1.084–1.357; p = 7.44 × 10^-4; n = 1406, 786 events; Table 5). In the subtype-, clinical-, and CNA-adjusted model, low hypoxia remained associated with longer survival time (TR = 1.198, 95% CI 1.070–1.342; p = 1.79 × 10^-3; Table 5), whereas CNA burden did not show an independent association with outcome (TR = 0.820, 95% CI 0.623–1.079; p = 0.157; Table 5).

**Table 5:** METABRIC validation of associations among the 16-gene hypoxia score, CNA burden, and overall survival.

METABRIC (n = 1979; events = 1143)	Result
Cox (descriptive): low vs high hypoxia	HR = 0.703 (95% CI 0.626–0.790), p = 3.27e-09
Cox (descriptive, subtype-adjusted): low vs high hypoxia	HR = 0.740 (95% CI 0.655–0.838), p = 1.79e-06
CNA burden difference (high vs low hypoxia)	Wilcoxon p = 2.15e-30
Weibull AFT (preferred, clinical-adjusted): low vs high hypoxia	TR = 1.213 (95% CI 1.084–1.357), p = 7.44e-04
Weibull AFT (preferred, subtype + clinical + CNA): low vs high hypoxia	TR = 1.198 (95% CI 1.070–1.342), p = 1.79e-03
Weibull AFT (preferred, subtype + clinical + CNA): CNA burden	TR = 0.820 (95% CI 0.623–1.079), p = 0.157

Note: Cox results are shown descriptively; because key adjusted METABRIC Cox models violated the proportional hazards assumption, Weibull AFT estimates were used as the preferred adjusted effect estimates.

The METABRIC results reproduced the direction of association among higher hypoxia, higher CNA burden, and poorer survival. Because key Cox models violated the PH assumption, the preferred adjusted survival estimates were interpreted in the Weibull AFT framework rather than the Cox framework.

### Summary of cross-cohort findings

Across TCGA and METABRIC, higher hypoxia was consistently associated with higher CNA burden. The survival association was more context-dependent. In TCGA, the cohort-wide association weakened after adjustment for subtype and clinical covariates. The clearest prognostic signal was observed in Luminal B tumors. This signal persisted after parsimonious clinical adjustment, although the Luminal B analysis included a limited number of events.

In METABRIC, high hypoxia was also associated with poorer survival after clinical and subtype adjustment. However, key Cox models violated the proportional hazards assumption. Therefore, the preferred adjusted survival estimates were interpreted in the Weibull AFT framework rather than the Cox framework. Across the final adjusted survival models, CNA burden did not show an independent association with outcome.

## Discussion and conclusions

By integrating transcriptomic hypoxia measures with global CNA burden, this study identified intrinsic subtype composition as an important source of confounding in pooled analyses of hypoxia and prognosis. In TCGA, a cohort-wide median split of the BUFFA hypoxia score separated survival groups. However, this signal largely reflected subtype imbalance. After adjustment for subtype and clinical covariates, the pooled TCGA association was no longer independently associated with OS.

Subtype-specific analysis showed heterogeneity in the hypoxia-survival association. The strongest signal was observed in Luminal B tumors. In this subtype, low hypoxia remained associated with better survival after adjustment for age and stage. This association also persisted after additional adjustment for CNA burden. By contrast, CNA burden itself was not independently associated with OS.

Across both cohorts, higher hypoxia was consistently associated with higher CNA burden. This supports a consistent association between hypoxic tumor biology and genomic instability. This interpretation is broadly consistent with prior work showing that aneuploidy and chromosomal instability are associated with adverse tumor phenotypes across cancers [27]. TP53 alterations were also enriched in hypoxia-high Luminal B tumors. This finding is biologically plausible because p53 pathway dysfunction has a central role in genomic stress responses and tumor progression [28].

However, CNA burden did not show an independent association with outcome in the final adjusted survival models. Its prognostic interpretation should therefore remain cautious. The Luminal B findings should also be interpreted cautiously because they were based on a relatively small number of events. These results are hypothesis-supporting rather than definitive.

In METABRIC, hypoxia remained associated with poorer outcome after subtype and clinical adjustment. However, key adjusted Cox models violated the proportional hazards assumption. Therefore, the preferred estimates were obtained from Weibull AFT models. Taken together, these findings support subtype-dependent prognostic relevance of hypoxia. They also support a directionally consistent association between hypoxia and genomic instability. However, they do not establish a uniform independent prognostic role for CNA burden across cohorts.

Several limitations should be considered. First, this was an observational study based on retrospective public-cohort data. The results therefore support association rather than causation. Second, the revised survival models incorporated subtype and available standard clinical covariates, but residual confounding cannot be excluded. Clinical completeness differed across cohorts. Some variables, especially treatment-related annotations, were not uniformly available or equally detailed in all analytic subsets.

Third, hypoxia and CNA burden were defined differently in TCGA and METABRIC. Cross-cohort comparisons should therefore be interpreted mainly in terms of directional consistency. Direct numerical equivalence of effect size or scale should not be assumed. Fourth, the key TCGA Luminal B findings were based on a limited number of events. This may reduce precision and the stability of multivariable estimates.

Finally, TP53 enrichment in hypoxia-high Luminal B tumors was biologically plausible and statistically robust within TCGA. However, this gene-level enrichment analysis was not independently replicated in a second cohort with comparable mutation and hypoxia data. These findings support a subtype-aware interpretation of hypoxia in breast cancer and show a consistent association between hypoxia and CNA burden across cohorts. Further validation in clinically annotated datasets is needed before these associations can be considered definitive.

## Supporting information

S1 File Reproducibility package containing processed TCGA and METABRIC analysis datasets, analysis code, the dataset manifest, session information, and final analysis output summaries. (ZIP)

S1 Table Summary of adjusted survival models, covariates included, complete-case sample sizes, and event counts. (XLSX)

S2 Table Proportional hazards diagnostic results for Cox models and identification of models for which Weibull accelerated failure time estimates were used as the preferred effect estimates. (XLSX)
